# Extrinsic grouping factors in motion-induced blindness

**DOI:** 10.1371/journal.pone.0192133

**Published:** 2018-01-30

**Authors:** Dina Devyatko, Alexander Pastukhov

**Affiliations:** 1 Laboratory for Cognitive Research, National Research University Higher School of Economics, Moscow, Russia; 2 Institute of Information Processing and Decision Making, University of Haifa, Haifa, Israel; 3 Department of General Psychology and Methodology, University of Bamberg, Bamberg, Bavaria, Germany; 4 Forschungsgruppe EPÆG (Ergonomics, Psychological Æsthetics, Gestalt), Bamberg, Bavaria, Germany; University of Melbourne, AUSTRALIA

## Abstract

We investigated how various grouping factors altered subjective disappearances of the individual targets in the motion-induced blindness display. The latter relies on a moving mask to render highly salient static targets temporarily subjectively invisible. Specifically, we employed two extrinsic grouping factors, the connectedness and the common region, and examined whether their presence would make targets more resilient against the suppression. In addition, we investigated whether the presence of an illusory Kanizsa triangle would affect the suppression of the inducing Pac-Man elements. We quantified the perceptual dynamics using the proportion of the disappearance time (this indicates whether targets became more resilient against the suppression), and the proportion of simultaneous disappearance and reappearance events (characterizes the tendency for the targets to disappear or reappear as a group). We report that a single mask that encompassed all targets (a common region grouping) significantly increased the proportion of simultaneous disappearance and reappearance events, but had no effect on the proportion of the disappearance time. In contrast, a line that connected two targets significantly decreased the total invisibility time, but had no impact on the simultaneity of the disappearance and reappearance events. We found no statistically significant effect of the presence of the illusory Kanizsa triangle on either measure. Finally, we found no interaction either between the common region and the connectedness or between the common region and the presence of the illusory Kanizsa triangle. Our results indicate that extrinsic grouping factors might influence the perception differently than the intrinsic ones and highlight the importance of using several measures to characterize the perceptual dynamics, as various grouping factors might affect it differentially.

## Introduction

We experience the world as being composed of objects, object parts, textures, etc., without noticing the clutter and ambiguity of retinal inputs. The visual system constructs the representation by structuring the visual scene into individual objects. This process of grouping is critical to the emergence of the object perception and it relies on various heuristics. The initial proposal by Gestalt psychologists included such grouping principles as proximity and similarity [1,2], and the original list has been greatly expanded and elaborated over the years (for the review please see [3]). Studies of grouping principles helped us to better understand the neural basis of the figure-ground segregation [4,5], as well as the development of perceptual grouping [6,7], and remain a valuable experimental tool.

In the real-world visual scene, multiple and, sometimes, conflicting grouping cues are often present simultaneously. A prime example is an animal or man-made camouflage that makes an animal blend into the environment (think polar bears in snow) or breaks up the silhouette (think zebras) [1,2]. This intrinsic complexity of a typical real-world scene prompted multiple studies that explored perceptual effects of grouping by multiple factors [8–14]. Furthermore, whenever multiple grouping factors are present, they can interact either enhancing or diminishing their effectiveness (for review see [15]). For example, Shibata et al. demonstrated an interaction between proximity and closure [13]. Other studies showed an interaction between the common region [16], on the one hand, and proximity and similarity [14], on the other.

Grouping factors can be classified into intrinsic and extrinsic. This differentiation was proposed by Palmer, who suggested connectedness and common region differ from the classical Gestalt principles and should be considered extrinsic [16,17]. Specifically, a connecting line between the two dots [18] or a contour that encompasses them [16] are not intrinsic objects’ properties and, therefore, group them by a virtue of providing an external context. Based on this distinction, most classical Gestalt grouping principles are intrinsic, as they rely on the intrinsic relationships between properties or features of the discrete elements, such as their color or shape.

**Fig 1:**
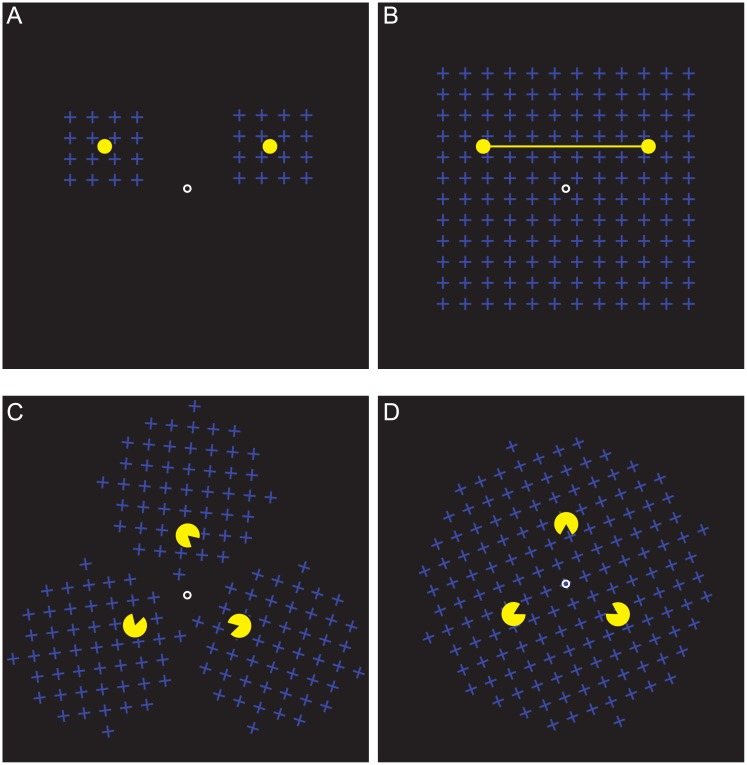
Experimental displays. A, B) Experiment 1. Targets were either (A) unconnected or (B) connected via a yellow line. Two types of masks were used: (A) two identical spatially separated masks, each covering a single target, or (B) a single common mask, which covered both targets. C, D) Experiment 2. Targets were Pac-Men figures that were either (C) misaligned (rotated 45° counterclockwise) or (D) aligned to facilitate perception of the illusory Kanizsa triangle. Two types of masks were used: (C) three rotating masks, each encompassing a single target, or, (D) a single common rotating mask, which enveloped all targets.

Methodologically, the strength of various grouping factors or of their combination can be assessed by examining whether grouped elements better resist the perceptual suppression from binocular rivalry, flash-induced perceptual fading or motion-induced blindness (MIB) than ungrouped ones [13,19–21]. The latter suppression method uses a moving mask, such as a grid of rotating crosses depicted in Fig 1, that renders highly salient static targets temporarily subjectively invisible [22]. MIB was used to explore the role of perceptual grouping in the formation of object representations [20,23–25] and to examine the interaction between various grouping factors [13]. Prior work using MIB indicates that grouping targets by the connectedness and the common region [20] as well as by proximity, good continuation, closure, and similarity [13,20,22] leads to the reduced suppression and synchronized disappearances of targets. However, semantic grouping appears to have no effect on rates of simultaneous disappearances for letter targets [26].

Here, we investigate the two extrinsic grouping factors proposed by Palmer: The common region and the connectedness [17]. In the former case, we manipulated the number of MIB masks. Either a common region was produced by a single mask that encompassed all targets or each target was paired with its own mask (Fig 1A and 1C versus Fig 1B and 1D). In the latter case, the two dots were presented in isolation or were connected by a line, which grouped them into a single object (Fig 1A versus Fig 1B). The use of two extrinsic grouping factors also allowed us to extend prior work on the interaction between intrinsic [13] and between extrinsic and intrinsic grouping factors [10,14].

In addition, we investigated whether the perception of an illusory Kanizsa triangle [27] is accompanied by a perceptual grouping of the inducing Pac-Man elements. In Kanizsa illusion, Pac-Man shapes induce the perception of an object that occludes the circles (Fig 1D). The illusion disappears when Pac-Man shapes are misaligned (Fig 1C). Prior work indicates that symmetry, closure and good continuation between Pac-Man targets influence the perception of the illusory Kanizsa triangle [28,29]. Because the previous study hints that the presence of the illusory figure affects grouping [30], we were curious whether it would alter Pac-Man shapes resistance against the perceptual suppression as well. To this end, we paired it with the extrinsic grouping by the common region, described above.

Below we present the results of three experiments that paired the common region and the connectedness extrinsic grouping factors (Experiment 1) and the common region with the illusory Kanizsa figure (Experiment 2 and 3). In all three experiments, we investigated how the presence of the individual grouping factors or of their combination alters the dynamics of perceptual disappearances using the MIB paradigm.

## Materials and methods

### Observers

Procedures were in accordance with the Declaration of Helsinki and were approved by the Institutional Review Board of National Research University “Higher School of Economics” (please refer to S1 File). All observers had normal or corrected-to-normal vision. Apart from the first author, observers were naïve as to the purpose of the experiments.

### Apparatus

The displays of Experiments 1 and 2 were presented on a 17” CRT screen Samsung SyncMaster 757 DFX, with a spatial resolution of 1280x1024 and the refresh rate of 85Hz, with one pixel subtending approximately 0.026° at a viewing distance of 57 cm. The displays of Experiment 3 were presented on a 21.5” LCD screen Samsung S22C450MW, with a spatial resolution of 1680х1050 and the refresh rate of 85Hz, with one pixel subtending approximately 0.024° at a viewing distance of 57 cm.

### Experiment 1

#### Observers

Fifteen observers (five females, age 17–26), including the first author, participated in the experiment.

#### Display

Two types of the mask were used, either two spatially separated masks (1.9° x 1.9°, Fig 1A) or a single common mask (6° x 6°, Fig 1B). Mask elements were blue crosses (luminance 1.13 cd/m^2^) and had linear dimensions of 0.4° x 0.4°. The masks rotated clockwise with an angular speed of 240°/sec. The rotation of the separate masks was synchronized.

Two targets (diameter 0.33°, luminance 32 cd/m^2^) were presented 1° above the fixation and at 2° to the side. The targets were either unconnected (Fig 1A) or connected with a yellow line (Fig 1B, length 3.6°, width 0.026°, luminance 32 cd/m^2^).

#### Procedure

Four display configurations were used in Experiment 1: Two mask conditions x two connectedness conditions. Observers were instructed to fixate on the central marker while attending to the yellow targets. They were asked to continuously press a key if the corresponding target was invisible (each target had a designated key). Each condition was presented for two minutes once throughout the experimental session. To familiarize participants with the task, the displays were shown informally before the experiment to ensure that they perceive the subjective disappearance of the targets and understand the response mapping.

#### The preliminary fingers-press asynchrony measurement

Each observer performed a prior control experiment with a single stationary mask and targets that physically disappeared from the screen. The number of disappearing targets (one or both) and the duration of the disappearance episode (between 1.5 and 3 seconds, full visibility episodes were between 2 and 4 seconds) were randomized. Observers performed two trials (one with connected targets, one with disconnected targets), each two-minutes long. We used results of individual observers to estimate distributions of the finger-press asynchrony measure (FPA) for cases of two targets disappearing simultaneously. In the main experiment, multiple consecutive key presses or releases were labeled as “simultaneous”, if the time window of their occurrence was between the minimal and maximal key press/release times in the control experiment.

### Experiment 2

#### Observers

Twenty-two observers (14 female; age 20–30), including the first author, participated in the experiment. The first author was the only participant who also participated in Experiment 1. Four additional observers were excluded during the preliminary testing because the MIB display was not effective (less than five total individual disappearance events per condition).

#### Display

Two types of masks were used, either three spatially separated masks (diameter 6°, Fig 1C) or a single common mask (diameter 10° x 6°, Fig 1D). Mask elements were blue crosses (luminance 1.13 cd/m^2^) and had linear dimensions of 0.4° x 0.4°. The masks rotated clockwise with an angular speed of 180°/sec. The rotation speed was lower than in Experiment 1 due to participants reporting an unpleasant perception when three masks were employed. The rotation of the separate masks was synchronized.

Three Pac-Man targets (diameter 0.72°, luminance 32 cd/m^2^, the diameter of a protection zone 0.8°) were presented at 2° of eccentricity. The targets were either misaligned (rotated 45° counterclockwise, Fig 1C) or aligned to facilitate the perception of the illusory Kanizsa triangle (Fig 1D).

#### Procedure

Four conditions were used in Experiment 2: Two mask conditions x two alignment conditions. Observers were instructed to fixate on the central marker while attending to the yellow targets. They were asked to continuously press a key if the corresponding target was invisible (each target had a designated key). Each condition was presented twice throughout the experimental session. First, it was presented for 30 seconds to familiarize observers with the display (trials 1–4), then again for two minutes, as part of the main experiment (trials 5–8). The analysis was carried out only on trials 5–8.

#### The preliminary fingers-press asynchrony measurement

Before the experiment, each observer performed a control experiment with a single stationary mask and targets that physically disappeared from the screen. The number of disappearing targets and the duration of the disappearance episode (between 1.5 and 3 seconds, full visibility episodes were between 2 and 4 seconds) were randomized. Observers performed two trials (trial duration was two minutes), one with the misaligned targets, one with the aligned targets. We used results for individual observers to estimate the distributions of the finger-press asynchrony measure (FPA) for cases of two and three targets disappearing simultaneously. In the main experiment, multiple consecutive key presses or releases were labeled as “simultaneous”, if the time window of their occurrence was between the minimal and maximal key press/release times in the control experiment.

### Experiment 3

#### Observers

Fourteen observers (ten females, age 21–51) participated in the experiment. Nine of them also participated in Experiment 2.

#### Display

Displays were identical to those used in Experiment 2, but only two conditions with the aligned targets were used (two trials each).

#### Procedure

Each condition was presented twice. Observers were instructed to press and hold the left arrow key if they perceived the illusory Kanizsa triangle. In addition, they were asked to press and hold the right arrow key if at least one target was not visible.

### Analysis

#### Measures for Experiments 1–2

To quantify the effect of grouping on the temporal dynamics of MIB in Experiments 1 and 2, we computed two measures: The proportion of the disappearance time for all targets (P_DISAPPEAR_) and the proportion of the simultaneous disappearances and reappearances for all targets (P_SIM_), which were defined as follows.

$$P_{DISAPPEAR=\frac{T_{DISAPPEAR}}{T_{TRIAL}}},$$ (1)

where T_DISAPPEAR_ is the total reported time when at least one target was invisible, and T_TRIAL_ is the duration of the trial.

$$P_{SIM}=\frac{D_{SIM}+R_{SIM}}{D_{TOTAL}+R_{TOTAL}},$$ (2)

where D_TOTAL_ and D_SIM_ are, respectively, a total number of disappearance events and the number of simultaneous disappearance events. Similarly, R_TOTAL_ and R_SIM_ are, respectively, a total number of reappearance events and the number of simultaneous reappearance events. D_SIM_ and R_SIM_ were computed using the finger-press asynchrony measure of individual observers. Specifically, multiple consecutive key presses or releases were labeled as “simultaneous”, if the time window of their occurrence was between the minimal and maximal key press/release times in the preliminary finger-press asynchrony measurement.

Of the two measures, P_DISAPPEAR_ quantifies the strength of the suppression of the targets by the rotating masks or, conversely, the ability of the targets to resist that suppression. Prior work indicates that grouping reduces the total disappearance time by, presumably, increasing the resilience of the targets [13,20,22].

The proportion of the simultaneous disappearance and reappearance events P_SIM_ quantifies how often the targets disappear together, *i*.*e*. as a group. Accordingly, grouping should facilitate simultaneous disappearances and reappearances of the targets, increasing P_SIM_.

Note that the two measures are independent in that P_DISAPPEAR_ quantifies the duration of targets’ disappearance, whereas P_SIM_ characterizes how they disappear. Accordingly, various grouping factors might affect these two measures independently.

#### Measures for Experiment 3

To quantify the subjective visibility of the Kanizsa triangle, we computed the proportion of the Kanizsa’s visibility time as:

$$P_{KANIZSA}=\frac{T_{KANIZSA}}{T_{TRIAL}-T_{DISAPPEAR}},$$ (3)

where T_KANIZSA_ is the total reported time when the illusory Kanizsa triangle was perceived by observers, T_DISAPPEAR_ is the total reported time when at least one target was invisible, and T_TRIAL_ is the duration of the trial.

To quantify the effect of the number of masks on the temporal dynamics of MIB, we used the proportion of disappearance time for all targets (P_DISAPPEAR_), which was computed analogously to that in Experiments 1–2.

#### Statistical analysis

Statistical analysis was performed in R [31] using the BayesFactor package [32] for Bayesian repeated measures ANOVA, packages *lme4* [33] and *lmerTest* [34] for linear mixed model analysis, and package *ggplot2* [35] to generate figures. Prior to plotting, measures were adjusted for the repeated-measures design following [36].

## Results

### Experiment 1. Grouping via the connecting line and the single mask

In our first experiment, we investigated a combination of two grouping factors—the connectedness (two targets connected by a line) and the common region (a single mask that encompasses all targets)–on the perception of the targets in MIB (Fig 1A and 1B). The presence or absence of the individual factors in a two-by-two design yielded four conditions, which we ordered in Fig 1 based on their expected grouping. A display with no factors (the unconnected targets and the two masks) served as a baseline condition. A display with the two targets connected by a line superimposed on the two separate masks (connectedness only), as well as a display with the unconnected targets and the single common mask (the common region only), both had only a single grouping factor that influenced perception. Finally, the fourth display had both grouping factors: the connected targets and the single common mask that provided the common region.

To quantify the influence of grouping on the temporal dynamics of MIB perception, we used two measures: a proportion of the total disappearance time and a proportion of the simultaneous disappearance and/or reappearance events (see Materials and methods section above).

The proportion of the total disappearance time was measured as the proportion of time when at least one target was invisible. It is the key measure of the perceptual suppression, which was introduced in the original study on MIB [22], and is widely used in perceptual suppression studies [37–42]. It characterizes how well a particular visual configuration can resist the suppression and how the efficient grouping tends to reduce the total time of disappearance [13,22]. Based on previous studies, we expected that stronger grouping would make the targets more resilient, reducing the proportion of the disappearance time [13,22].

The second measure, the proportion of the simultaneous disappearance and/or reappearance events, shows whether all targets tend to disappear or reappear together as a group. Here, we expected that grouping would facilitate simultaneous events, whereas ungrouped targets should be more likely to disappear and to reappear at different times. Because these two measures quantify different aspects of perceptual dynamics, we also sought to establish how they reflect the influence of various grouping factors and whether observed changes in two measures are associated. To quantify the effect of grouping, we performed both multilevel linear mixed model [33,34] and a repeated measures Bayesian ANOVA [32] analyses.

**Fig 2:**
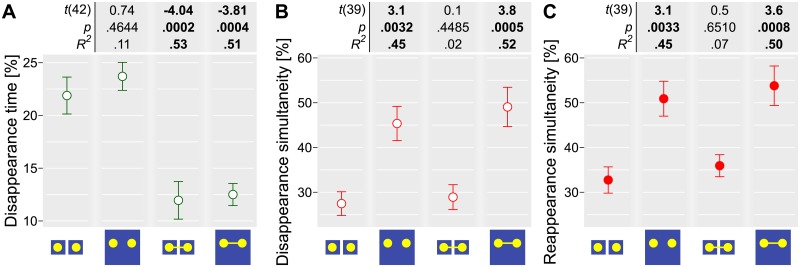
Experiment 1. The effect of the individual grouping factors and of their combination on the proportion of the disappearance time (A) and the proportion of the simultaneous disappearance (B) and reappearance (C) events. Circles and error bars depict, respectively, mean ± 1 S.E.M. Values above the plot depict the Student’s *t*-statistics (Satterthwaite approximations to degrees of freedom), the corresponding *p*-values, and effect sizes when comparing the corresponding condition to the baseline. The comparison was performed using a linear mixed-effects model analysis.

We found that the total disappearance time was significantly reduced by the presence of the connecting line, but was not affected by the number of masks (see Fig 2A and Table 1A). For the simultaneity of disappearance and reappearance events, the type of event had a significant effect (*χ*^2^(1) = 4.04, p = .0442, for the details of the analysis, please refer to the online repository). Therefore, we analyzed these two types of events separately. We found that in both cases the presence of the single mask, but not of the connecting line, strongly and significantly increased the proportion of simultaneous events (Fig 2B and 2C and Table 1B and 1C). Although reappearance events were generally more likely to occur simultaneously than disappearance ones, the relative effect of the factors was virtually identical. Finally, we did not observe an interaction between the factors for any measure.

**Table 1:** Statistical analysis using the multilevel linear mixed model and the repeated measures Bayesian ANOVA analyses. The effect of the number of masks and of the connecting line on (A) the disappearance time when at least one target invisible, (B) simultaneity of the disappearance events, and (C) simultaneity of the reappearance events. The random factor was participants’ identity. A,B) *df*: degrees of freedom. AIC: Akaike’s Information Criterion. The Bayes factor was computed relative to the model with random effects only. χ^2^ was computed relative to the preceding simpler model.

A) Disappearance time, at least one target invisible						
Model	*df*	AIC	Log Likelihood	χ^2^	*p*-value	Bayes Factor
Participant	3	-92.2	49.1			
+ Mask	4	-90.5	49.2	0.26	.6082	0.30 ± 1.16%
**+ Connection**	**5**	**-116.7**	**-126.7**	**28.30**	**< .0001**	17209.92 ± 1.23%
+ Mask: Connection	6	-114.9	-126.9	0.14	.7066	6314.71 ± 3.56%
B) Simultaneity of disappearance events						
Model	*df*	AIC	Log Likelihood	χ^2^	*p*-value	Bayes Factor
Participant	3	524.6	-259.3			
**+ Mask**	**4**	**508.2**	**-250.1**	**18.35**	**< .0001**	**691.92 ± 3.05%**
+ Connection	5	509.9	-249.9	0.34	.5607	208.20 ± 1.45%
+ Mask: Connection	6	511.8	-249.9	0.13	.7201	73.06 ± 2.53%
C) Simultaneity of reappearance events						
Model	*df*	AIC	Log Likelihood	χ^2^	*p*-value	Bayes Factor
Participant	3	524.1	-259.0			
**+ Mask**	**4**	**509.7**	**-250.9**	**16.32**	**< .0001**	**274.28 ± 0.83%**
+ Connection	5	511.2	-250.6	0.49	.4841	92.79 ± 2.99%
+ Mask: Connection	6	513.2	-250.6	0.00	.9951	33.43 ± 4.21%

### Experiment 2. Grouping via the Kanizsa figure and the single mask

The second experiment, again, compared influence of a grouping effect of a single mask to that of several masks. In addition, we changed the targets into Pac-Man elements (Fig 1C and 1D). When properly aligned (Fig 1D), they induce the perception of an illusory Kanizsa triangle. We were curious, whether such a contingent grouping by the virtue of being occluded by the same object would have a measurable effect on perception. As in Experiment 1, we were also interested whether the two factors would interact.

**Fig 3:**
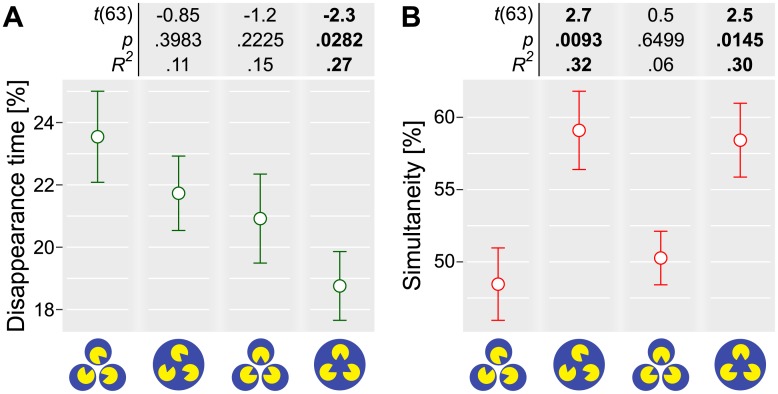
Experiment 2. The effect of the individual grouping factors and of their combination on the proportion of the disappearance time (A) and the proportion of the simultaneous disappearance and reappearance events (B). Circles and error bars depict, respectively, mean ± 1 S.E.M. Values above the plot depict the Student’s *t*-statistics (Satterthwaite approximations to degrees of freedom), the corresponding *p*-values, and effect sizes when comparing the corresponding condition to the baseline. The comparison was performed using a linear mixed-effects model analysis.

The presence or the absence of the individual factors in a two-by-two design yielded four conditions, which we ordered in Fig 3 based on their expected grouping strength. A display with both factors absent (the misaligned Pac-Man targets and the multiple masks) served as a baseline. Two display configurations had a single factor. A display with the aligned targets, which facilitated perception of the illusory Kanizsa triangle (the illusory triangle only), but with the multiple masks, and a display with the misaligned targets and the single common mask (the common region only). Finally, the fourth display had both factors (the aligned targets and the single common mask).

We used the same measures—the proportion of the disappearance time and the proportion of the simultaneous disappearance and reappearance events—and, again, we expected that grouping should decrease the former but increase the latter.

We found that the total disappearance time was significantly reduced only when both factors were present (see Fig 3A). Neither of the individual factors exerted significant influence alone, although the effect of the alignment was close to the significance level of .05 (see Table 2A). In contrast to Experiment 1, the type of the event (disappearance or reappearance) had no influence on the proportion of simultaneous events. Therefore, we analyzed all events together and included their type as an additional factor, alongside the number of masks and the alignment. We found that the number of masks, but not the targets’ alignment or the type of the event (disappearance or reappearance), had a strong and significant effect on the proportion of the simultaneous disappearance/reappearance events (see Fig 3B and Table 2B). Finally, we found no evidence of the interaction between two grouping factors for either measure (Table 2).

**Table 2:** Statistical analysis using the multilevel linear mixed model and the repeated measures Bayesian ANOVA analyses. A) Effect of the number of masks and of the targets’ alignment on the disappearance time when at least one target invisible. The random factor was participants’ identity. B) Effect of the number of masks, of the alignment, and of the event type (disappearance or reappearance) on the simultaneity of disappearance and reappearance events. The random factor was participants’ identity. A,B) *df*: degrees of freedom. AIC: Akaike’s Information Criterion. The Bayes factor was computed relative to the model with random effects only. χ^2^ was computed relative to the preceding simpler model.

A) Disappearance time, at least one target invisible						
Model	*df*	AIC	Log Likelihood	χ^2^	*p*-value	Bayes Factor
Participant	3	-160.8	83.4			
+ Mask	4	-160.5	84.2	1.70	.1919	0.46 ± 1.02%
+ Alignment	5	-162.0	86.0	3.52	.0606	0.46 ± 1.30%
+ Mask: Alignment	6	-160.0	86.0	0.01	.9059	0.14 ± 3.88%
B) Simultaneity of disappearance and reappearance events						
Model	*df*	AIC	Log Likelihood	χ^2^	*p*-value	Bayes Factor
Participant	3	-105.2	55.6			
**+ Mask**	**4**	**-121.6**	**64.8**	**18.45**	**< .0001**	**897.13 ± 1.37%**
+ Alignment	5	-119.7	64.8	0.07	.7900	148.25 ± 1.64%
+ Event type	6	-117.7	64.8	0.01	.9305	23.38 ± 2.00%
+ Mask: Alignment	7	-116.0	65.0	0.34	.5579	6.20 ± 3.64%
+ Mask: Event type	8	-115.1	65.5	1.03	.3093	2.01 ± 3.9%
+ Alignment: Event type	9	-113.1	65.6	0.07	.7861	0.45 ± 3.06%
+ Full interaction	10	-111.2	65.6	0.06	.7997	0.14 ± 3.92%

### Experiment 3. Visibility of the illusory Kanizsa triangle

In our second experiment, we tested whether the alignment of Pac-Man targets would alter targets’ ability to resist the perceptual suppression. The alignment should produce the perception of an illusory Kanizsa triangle (Fig 1D) that occludes all individual targets. Although we did not observe a significant effect of alignment, it is possible that our configuration simply failed to induce the illusory figure. Accordingly, in our third experiment, we explicitly tested whether observers perceived the illusory figure both when it was placed over a single mask and when separate masks were encompassing individual targets.

To this end, we repeated the experiment using two display configurations with the aligned targets but an altered response mapping. Specifically, the observers reported on the visibility of all targets, pressing the designated key if at least one of them became invisible, and on the visibility of the illusory Kanizsa triangle, pressing a different key if they perceived it. Because observers were using a single key to report on the visibility of all targets, we had no means to analyze the simultaneity of the targets’ disappearance or reappearance.

**Fig 4:**
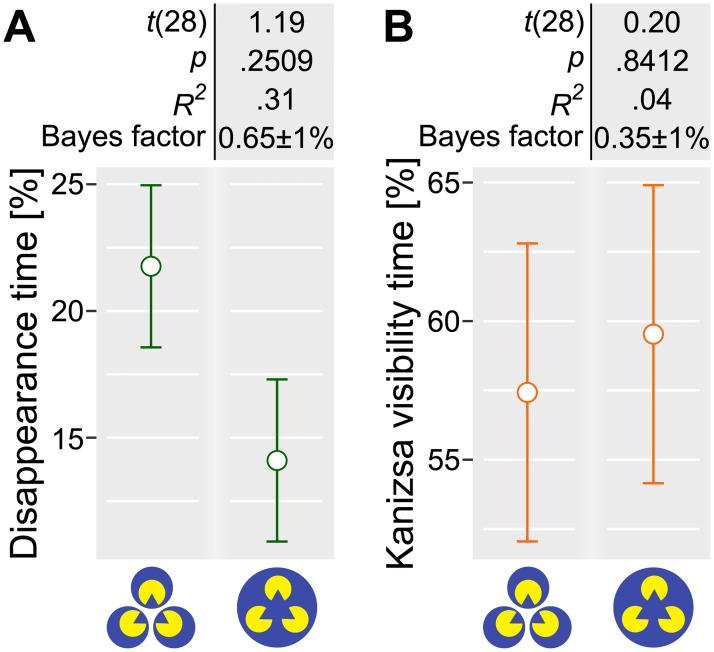
Experiment 3, the effect of the number of masks on the proportion of the disappearance time (A) and on the visibility of the illusory Kanizsa figure (B). Circles and error bars depict, respectively, mean ± 1 S.E.M. Values above the plot depict the Bayes factor, the Student’s *t*-statistics (Satterthwaite approximations to degrees of freedom), the corresponding *p*-values, and effect sizes when comparing the single mask condition to the baseline three masks condition. The comparison was performed using a linear mixed-effects model analysis.

With respect to the proportion of the time at least one target was invisible, results mirror those of Experiment 2. The proportion of the disappearance time decreased when a single mask was used, but this effect was not statistically significant (Fig 4A). Importantly, we found no effect of the masks on the subjective visibility of the illusory Kanizsa figure. In both conditions, it was perceived for approximately 60% of the time (Fig 4B). Thus, we conclude that the visibility of the Kanizsa figure in Experiment 2 was not compromised by the other grouping factor.

## Discussion

Here, we examined how perceptual grouping influences the dynamics of perceptual disappearances in motion-induced blindness (MIB). To this end, we employed two extrinsic grouping factors, the connectedness and the common region, and examined whether their presence would decrease subjective disappearances of the targets. In addition, we investigated whether the presence of the illusory Kanizsa triangle would perceptually group the inducing Pac-Man elements, again, affecting their subjective disappearance. The dynamics of subjective invisibility was quantified using two measures: The proportion of the disappearance time, which quantified whether grouping made targets more resilient against the suppression produced by the rotating masks, and the proportion of the simultaneous disappearance and reappearance events, which characterized the tendency for the targets to disappear or reappear together, i.e. as a group.

We report that the common region (a single mask that encompassed all targets) consistently and significantly increased the proportion of simultaneous disappearance and reappearance events. However, it had no effect on the overall proportion of the time the targets were invisible. The effect of the connecting line was exactly opposite. Although it significantly decreased the total invisibility time, it had no impact on the simultaneity of the disappearance and reappearance events.

The presence of the illusory Kanizsa triangle produced no statistically significant effect on either measure, although our results indicate that it might influence the proportion of the disappearance time (see Fig 3A). The weakness of this effect most likely stems from the fact that the grouping [43], and the illusory Kanizsa figures [44,45], may require visual awareness and, therefore, may break down during the perceptual disappearance. Finally, we found no interaction either between the common region and the connectedness or between the common region and the presence of the illusory Kanizsa triangle.

Our results add to the growing body of the literature on the interaction between perceptual grouping factors. The emergent picture indicates that its presence depends on the specific choice of grouping factors. On the one hand, we failed to see any evidence of the interaction between the chosen factors, mirroring the previous results on proximity and orientation similarity [13]. On the other hand, the interaction effects were demonstrated previously for the pairs of intrinsic grouping factors, as well as between intrinsic and extrinsic grouping factors [9,10,12–14]. This difference might reflect the different nature of the grouping factors (e.g., both intrinsic, an intrinsic and an extrinsic, or both extrinsic). Similarly, it is not presently clear whether the presence or the absence of the interaction can be used to differentiate between different types of the grouping factors. However, it might serve as a useful measure alongside other considerations.

Our results also highlight the importance of using several measures to quantify the influence of grouping factors. In our case, the common region and the connectedness both affected the dynamics of the perceptual suppression. However, the nature of their influence was very different. For example, it is possible that connecting the elements with a line produced a new object but did not influence the background. Conversely, a change in the number of masks could have altered the visual context but not the object itself. In other words, the observed differences might reflect that these two grouping manipulations operate at different representation levels. These differences are likely to be useful for creating a classification of the grouping factors, as well as in guiding the imaging studies. In particular, it would be interesting to investigate whether there are neural correlates specific to a particular measure but common across various grouping factors. This type of neural correlates would be similar to that found for perceptual suppression in multi-stable figures [46].

Finally, our Experiment 1 lends itself naturally to a comparison with an earlier study that also investigated the effect of connectedness in MIB [20]. In that study, at the beginning of a trial, two target dots were either connected by a line segment or disconnected from the line segment. When the dots and the line segment disappeared due to MIB, the line segment could either shrink or grow in order to change the object representation of the dots outside of awareness. This manipulation had a significant effect on the percentage of reports about simultaneous reappearances [20]. In contrast to the earlier report, we failed to observe any effect of the connecting line on the proportion of the simultaneous events. However, numerous methodological differences might explain this discrepancy. First, the analysis in the original work was limited only to the relatively long disappearance events (>1 second). Second, Mitroff and Scholl [20] used a retrospective verbal report, whereas we relied on the finger press asynchrony. Third, the rotation speed of the mask in our study was lower than the one used by Mitroff and Scholl (respectively, 240°/s and 470°/s), which could have affected both the frequency and duration of disappearances [22,47]. Finally, the effect observed in the original study could reflect specifics of the population sample, as it was rather small (five observers, though each of them completed six 8-minute long trials) and contained experienced observers, as opposed to fifteen novice observers who participated in our study. In other words, although it is very much possible that our methodology precluded us from observing the increase in the simultaneity of events, these results nonetheless indicate that the effect in question may be rather weak and may require specific experimental conditions to be reproduced.

## Conclusions

Our results, together with previous findings, indicate that extrinsic grouping factors might influence perception differently than the intrinsic ones. They also highlight the importance of using several measures to characterize the perceptual dynamics, as various grouping factors might affect it differentially.

## Supporting information

S1 File Devyatko and Pastukhov—Ethical approval. (PDF)
