# Supplementary material for: Extrinsic grouping factors in motion-induced blindness
Source: PLoS One. 2018 Jan 30;13(1):e0192133. doi: 10.1371/journal.pone.0192133 (PMC5790270; doi:10.1371/journal.pone.0192133)
Supplement: S1 File — (PDF) [file pone.0192133.s001.PDF]

National Research University Higher School of Economics

Statement of Opinion

on compliance of the Empirical Research Project with ethical norms

I hereby certify that the project titled "The Lack of Interaction between Extrinsic Grouping Factors in Motion-Induced Blindness" (Dina Devyatko, research fellow, Laboratory for Cognitive Research, HSE) was reviewed by the Institutional Review Board of the National Research University Higher School of Economics (HSE IRB) at its meeting on February 07, 2017 in line with the procedure established at the university. Based on the vote held by its members, the IRB decision is approved as follows:
The empirical research project fully complies with the ethical norms.

SIGNATURE: ______________________

Professor V.V.Radaev

Chair of the HSE Institutional Review Board
HSE First Vice-rector

Date: February 07, 2017
